# Supplementary material for: Fertility Gene Introns Harbor Transposable Elements that Shape Y-Loop Architecture
Source: bioRxiv. 2026 Jul 12:2026.07.08.737335. Preprint. [Version 1] doi: 10.64898/2026.07.08.737335 (PMC13370334; doi:10.64898/2026.07.08.737335)
Supplement: 1 [file NIHPP2026.07.08.737335v1-supplement-1.pdf]

bioRxiv preprint doi: https://doi.org/10.64898/2026.07.08.737335; this version posted July 12, 2026. The copyright holder for this preprint (which was not certified by peer review) is the author/funder, who has granted bioRxiv a license to display the preprint in perpetuity. It is made available under a CC-BY 4.0 International license.

Figure S1

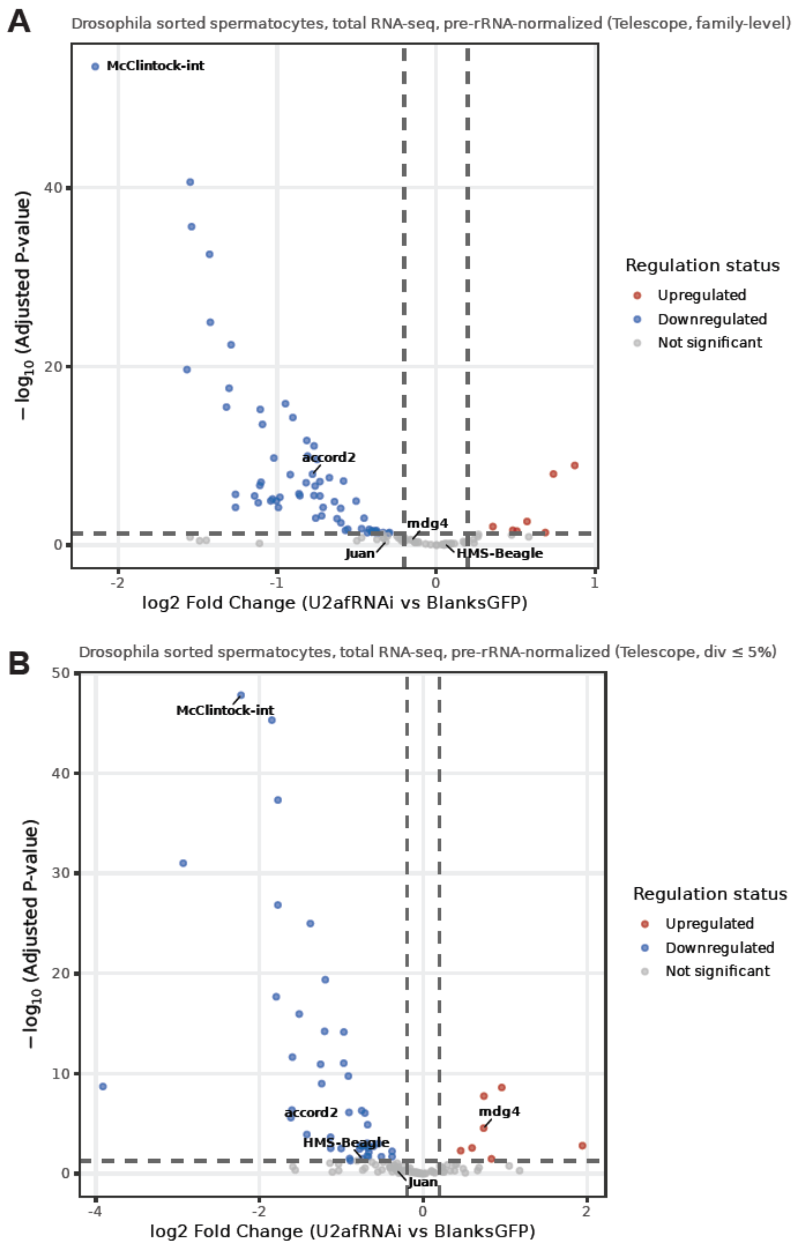

Figure S1. Telescope based quantification of TE expression

(**A-B**) Volcano plot showing DESeq2 analysis of RNA-seq data from isolated SCs comparing control (*bamGal4*, Blanks-GFP) and U2af38 knockdown (*bamGal4* >U2af38 RNAi, Blanks-GFP) testes using Telescope. Features were colored by regulation status: upregulated (adjusted p-value < 0.05, log2 fold change > 0.2), downregulated (adjusted p-value < 0.05, log2 fold change < −0.2), or not significant. Data shown in these plots are provided in Spreadsheet 3. In B, TE aligned reads whose divergence from consensus exceeded 5% were excluded.

bioRxiv preprint doi: https://doi.org/10.64898/2026.07.08.737335; this version posted July 12, 2026. The copyright holder for this preprint (which was not certified by peer review) is the author/funder, who has granted bioRxiv a license to display the preprint in perpetuity. It is made available under a CC-BY 4.0 International license.

Figure S2

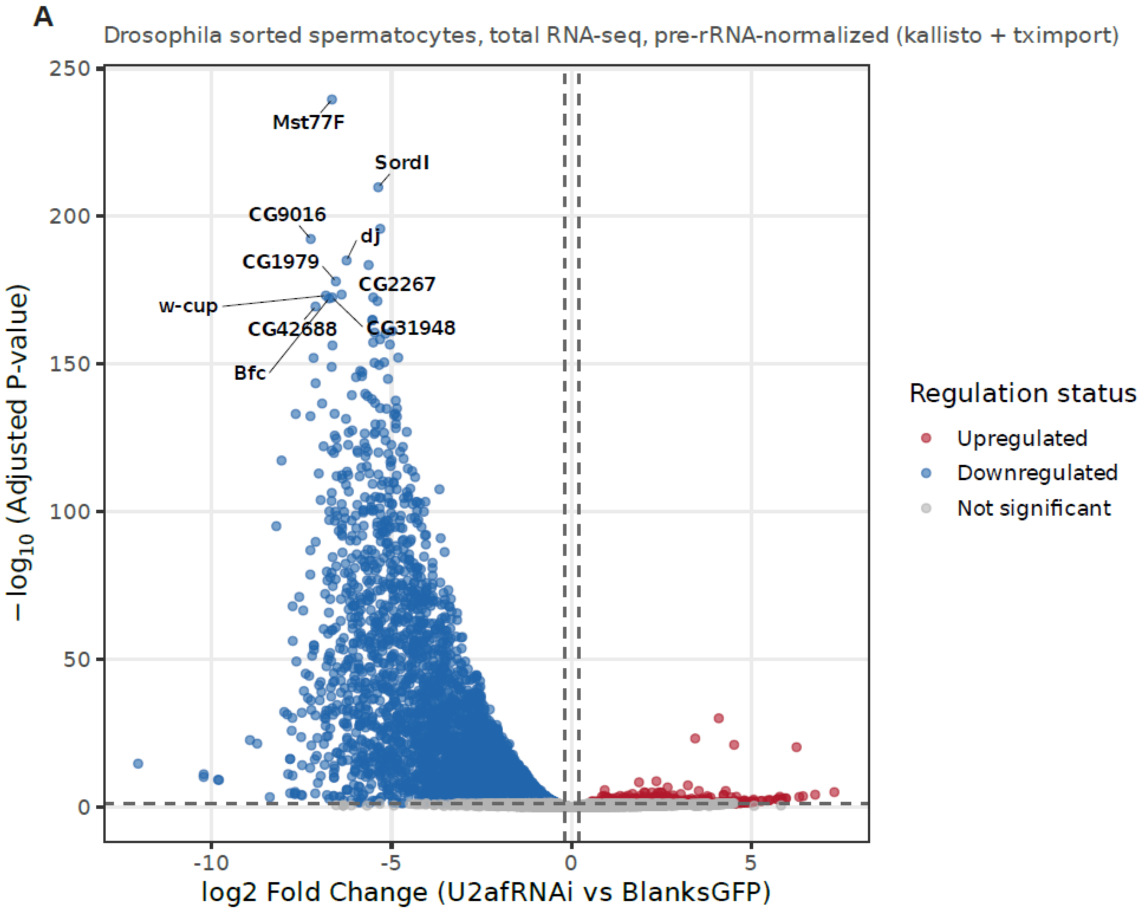

Figure S2. Gene expression changes in SCs from U2af38 knockdown testes.

Related to Figure 3L. (**A**) Volcano plot showing DESeq2 analysis of RNA-seq data from isolated SCs comparing control (*bamGal4*, Blanks-GFP) and U2af38 knockdown (*bamGal4* >U2af38 RNAi, Blanks-GFP) testes using featureCounts. Features were colored by regulation status: upregulated (adjusted p-value < 0.05, log2 fold change > 0.2), downregulated (adjusted p-value < 0.05, log2 fold change < −0.2), or not significant. Only genes are shown in this volcano plot. Data shown in these plots are provided in Spreadsheet 3.
